# Correction: Neuromuscular coordination of movement and breathing forges a hammer-like mechanism for woodpecker drilling

**DOI:** 10.1242/jeb.252195

**Published:** 2026-02-02

**Authors:** Nicholas D. Antonson, Stephen Ogunbiyi, Margot Champigneulle, Thomas J. Roberts, Franz Goller, Matthew J. Fuxjager

There was an error in *J. Exp. Biol.* (2025) 228, jeb251167 (doi:10.1242/jeb.251167).

During preparation of the figures for publication, after peer review, the authors inadvertently used an incorrect version of Fig. 2D that duplicated one of the bar graphs. Specifically, the purple bar graph representing the iliotibialis cranialis (IC) muscle is a duplicate of the blue bar graph representing the musculus depressor caudae (MDc) muscle. The corrected and original panel are shown below.

**Fig. 2D (corrected panel).**
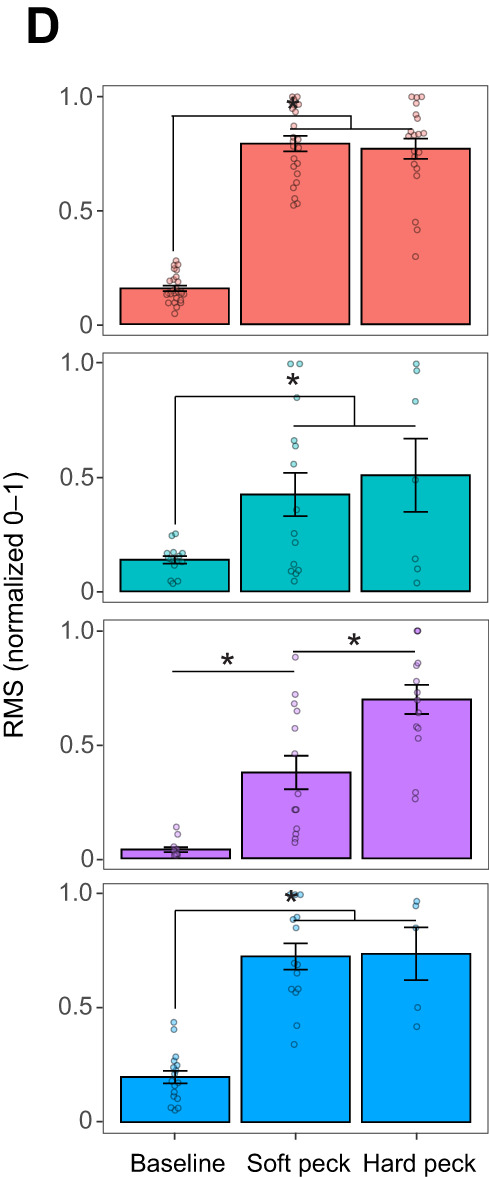
**Patterns of muscle activation during soft peck drilling behavior.** (D) Variation in EMG intensity when birds perform soft versus hard pecks. C and D show estimated marginal means±1 s.e.m. Asterisks denote significant differences between groups (*P<0.05). Individual data points (replicates) are shown with opaque circles for *n*=4 birds for LCv and MC and *n*=3 birds for the LCd, FCl, MOEa, IC, IF and MDc.

**Fig. 2D (original panel).**
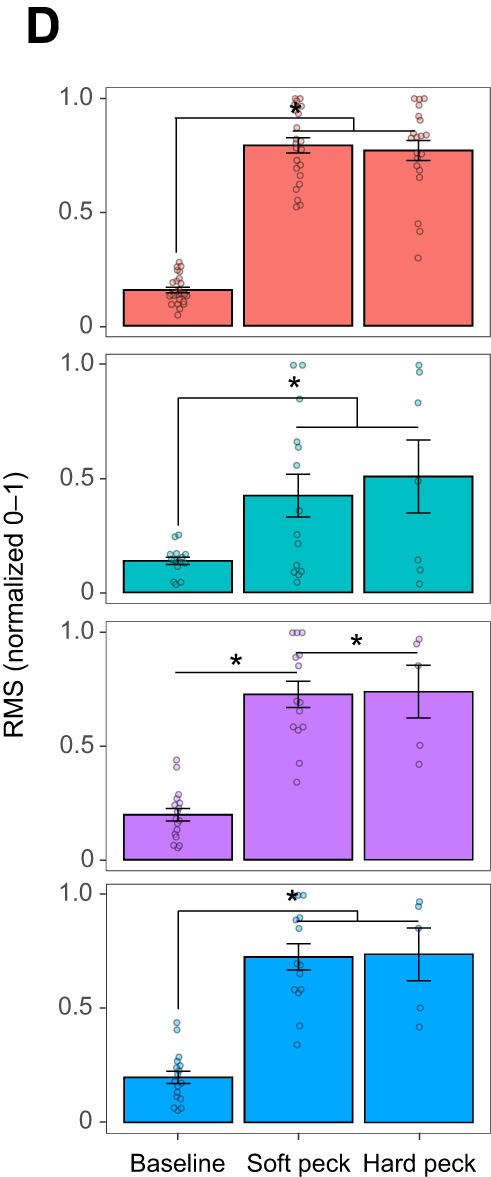
**Patterns of muscle activation during soft peck drilling behavior.** (D) Variation in EMG intensity when birds perform soft versus hard pecks. C and D show estimated marginal means±1 s.e.m. Asterisks denote significant differences between groups (*P<0.05). Individual data points (replicates) are shown with opaque circles for *n*=4 birds for LCv and MC and *n*=3 birds for the LCd, FCl, MOEa, IC, IF and MDc.

Both the online full-text and PDF versions of the article have been updated. The authors apologise to the readers for this error. This correction does not affect the results, interpretation or conclusions of the study and all analyses, code and statistics reported in the paper and supplementary materials are correct.

